# Quantitative fragment analysis of *FLT3*-ITD efficiently identifying poor prognostic group with high mutant allele burden or long ITD length

**DOI:** 10.1038/bcj.2015.61

**Published:** 2015-08-14

**Authors:** Y Kim, G D Lee, J Park, J-H Yoon, H-J Kim, W-S Min, M Kim

**Affiliations:** 1 Department of Laboratory Medicine, College of Medicine, The Catholic University of Korea, Seoul, Republic of Korea; 2 Catholic Genetic Laboratory Center, College of Medicine, Seoul St Mary's Hospital, The Catholic University of Korea, Seoul, Republic of Korea; 3 Cancer Research Institute, Division of Hematology, Department of Internal Medicine, Catholic Blood and Marrow Transplantation Center, College of Medicine, The Catholic University of Korea, Seoul, Republic of Korea

## Abstract

Mutation of the fms-like tyrosine kinase 3-internal tandem duplication (*FLT3*-ITD), which is one of the most frequent genetic alterations, strongly contributes to an increased risk of treatment failure and to poor prognosis. In this study, we established quantitative fragment analysis of *FLT3*-ITD simultaneously measuring mutant allele burden and length, verified the analytical performance and evaluated the clinical significance in adult acute myeloid leukemia (AML) patients. *FLT3-*ITD was detected in 73 of 363 adult AML patients (20.1%) and high mutant allelic burden (⩾50%, *n*=13) and long ITD length (⩾70 base pairs, *n*=15) were significantly associated with inferior overall survival (OS; *P*=0.002 and 0.005, respectively) and event-free survival (EFS; *P*=0.004 and 0.007, respectively). *FLT3*-ITD poor prognostic group was identified as patients with high allele burden or long ITD length (*n*=24), which revealed significant adverse clinical outcome for both OS (*P*<0.001) and EFS (*P*<0.001). In cytogenetically normal AML, even *FLT3*-ITD low allele burden and short length was associated with poorer OS (*P*=0.037) and EFS (*P*=0.044) than wild type, whose influence was overcome when hematopoietic stem cell transplantation was performed. In minimal residual disease monitoring, *FLT3-*ITD negativity after consolidation therapy was a valuable predictor of better OS (*P*<0.001) and EFS (*P*<0.001). *FLT3-*ITD poor prognostic group with high mutant allele burden or long ITD length is efficiently identified by quantitative fragment analysis.

## Introduction

Mutation of the fms-like tyrosine kinase 3 gene consisting of internal tandem duplication (*FLT3*-ITD), which is one of the most frequent genetic alterations, occurs in 15–35% of adults with acute myeloid leukemia (AML).^1, 2^ Many studies have reported that the *FLT3*-ITD mutation strongly contributes to an increased risk of treatment failure and to a poor prognosis.^3, 4^ Detection of ITD mutations at diagnosis is now a routine clinical practice to provide guidance of optimal treatment for AML patients. Currently, most AML patients with ITD mutations need hematopoietic stem cell transplantation (HSCT).^5, 6^

*FLT3*-ITD mutations are amenable to PCR-based molecular diagnostic DNA testing because they are limited to a small, predictable region of the *FLT3* gene. PCR amplification followed by fragment analysis is a rapid, sensitive and specific method that is commonly used. This method is able to detect mutant allele burden and changes in the length of ITD simultaneously.

Despite the evident poor outcome associated with the *FLT3*-ITD mutation, it is unclear whether this can be explained simply by the presence or absence of the abnormality, or whether other factors including mutant allele burden or mutant length influence its prognostic impact. A high total mutant level (more than 50%) revealed significantly worse clinical outcome.^7^ A recent study showed that minor mutants with a low allele burden can be clinically significant because they may become a dominant clone at the later refractory status.^8^ Persistence of *FLT3*-ITD minimal residual disease (MRD) after induction chemotherapy are predictive of complete remission duration.^9^ But in another study *FLT3* allele burden had no significant influence on outcomes after correcting for other variables.^10^ The meaning of the length of *FLT3*-ITD is also still debatable. In one study, mutant length and number had no significant impact on outcome,^7^ whereas others reported that patients with shorter ITDs have a lower relapse rate and more favorable outcome than those with longer ITDs.^11, 12, 13^ It is therefore necessary to clarify the significance of those indicators to determine the ideal treatment modality in AML with *FLT3*-ITD.

In this study, we measured the relative mutant allele burden and length of *FLT3*-ITD using fragment analysis and verified the analytical performance. The clinical significance was investigated in ITD mutants with allele burden and length to define risk groups in adult AML.

## Materials and Methods

### Patients and samples

Adult patients (*n*=363) diagnosed with *de novo* AML in Seoul St Mary's Hospital were included in the study. Diagnosis of AML was determined according to the 2008 World Health Organization classifications. The median age of 191 males and 172 females was 51 years (range, 15–85 years). Median follow-up was 31 months from initial presentation (range, 11.3–56.6 months). Patients were immunophenotyped using flow cytometric analyses. Cytogenetic analysis was performed using bone marrow (BM) specimen and classified based on National Comprehensive Cancer Network 2014 guidelines. Serial follow-up BM samples after treatment were available from 42 patients whose *FLT3*-ITD results were positive at diagnosis. DNA isolated from the peripheral blood or BM of 50 normal donors was used to analyze method specificity. According to the Declaration of Helsinki, all patients and donors in this study provided written informed consent for genetic analyses. The study protocol was approved by the Institutional Review Board of Seoul St Mary's Hospital, The Catholic University of Korea.

### Fragment analysis for *FLT3*-ITD

Genomic DNAs were extracted from the BM aspirates with the QIAamp DNA Mini Kit (Qiagen, Hamburg, Germany). The DNA was quantified spectrophotometrically using a ND-1000 apparatus (Nanodrop Technologies, Wilmington, DE, USA). PCR for fragment analysis to detect an ITD mutation was performed using a modified protocol based on that previously described.^14^ The functional domains of *FLT3* gene (Gene Bank Accession NM_004119.2) were PCR-amplified with forward primers 5′ end labeled with fluorescent dye. The PCR products were analyzed using a model 3130XL genetic analyzer (Applied Biosystems, Foster City, CA, USA) and the amplicons with a size greater than that of wild type (328±1 bases) were interpreted as positive for the ITD mutation. The number, area and length of mutant peaks on the electropherogram were analyzed using GeneMapper analysis software (Applied Biosystems). The *FLT3*-ITD mutant allelic burden was calculated as the ratio of the area under the curve of mutant and wild-type alleles (mutant/total *FLT3*). All tests were duplicated. Specificity was evaluated using DNA samples derived from 50 normal donors. No false positivity was identified. Mutant plasmid DNA was prepared by cloning and the detection sensitivity was defined as 10 copies of mutant plasmid DNA. Analytical sensitivity was analyzed using serial mixtures of mutant and wild-type plasmid DNA. Limit of detection was calculated by Probit analysis at 95% detection level as 3% of mutant allele burden. A strict linear correlation was observed with correlation coefficient (*r*^2^) of 0.99.

### Treatment courses

Of the 363 patients, 30 opted for conservative treatment, and 333 were treated according to our standard induction chemotherapeutic regimens. Briefly, 152 (45.6%) were treated with 3+7 idarubicin (IDA) plus *N*_4_-behenoyl-1-β-d-arabinofuranosyl cytosine (BHAC)^15^ as remission induction chemotherapy. IDA was administered daily at a dose of 12 mg/m^2^ intravenous for 3 consecutive days, and BHAC was administered daily at 300 mg/m^2^ for 7 consecutive days. A total of 137 patients (41.1%) were treated with 3+7 IDA plus cytosine arabinoside (ARA-C) at a dose of 100 mg/m^2^. Forty four patients (13.2%) were treated with modified low-dose ARA-C (20 mg/m^2^ every 12 h) combined with oral etoposide, 100 mg for 14 consecutive days.^16^

A total of 245 patients (67.5%) achieved complete remission (CR) after first cycle of induction chemotherapy. After achievement of CR, one or two consolidation chemotherapy was administered, or patients who were not in CR after 1st induction chemotherapy were treated by re-induction chemotherapy. The standard consolidation chemotherapy consisted of ‘3+5' mitoxantrone (12 mg/m^2^ intravenous) plus intermediate-dose ARA-C (1.0 g/m^2^ intravenous b.i.d.) or IDA (12 mg/m^2^) plus intermediate-dose ARA-C, applied alternatively. Seventy six patients relapsed or died during chemotherapy and 257 patients received final treatment (that is, allo-HSCT, autologous (auto)-HSCT or at least two cycles of consolidation chemotherapy after induction chemotherapy). Among these 257 patients, 183 had variable allo-HSCT courses. Eighty eight patients with an available human leukocyte antigen-matched sibling donor and 36 patients with a partially matched (<2 allele mismatched) unrelated donor underwent allo-HSCT. Thirty six patients received a haploidentical familial mismatched transplant.

As a reduced-intensity conditioning regimen, we administered busulfex 6.4 mg/kg and fludarabine 150 mg/m^2^ with 400 cGy total body irradiation (TBI). The myeloablative conditioning regimen included cyclophosphamide 120 mg/kg combined with 1320 cGy TBI or busulfex 12.8 mg/kg. Familial mismatched transplant comprised busulfex 6.4 mg/kg, fludarabine 150 mg/m^2^ and 800 cGy TBI with antithymocyte globulin (1.25 mg/day) for 4 days.^17^ If a patient achieved CR and did not have a suitable donor, we also considered auto-HSCT in 15 young patients (age <60 years) with a myeloablative conditioning regimen consisting of ARA-C 9 g/m^2^, melphalan 100 mg/m^2^ and 1200 cGy TBI.^18^ The remaining 55 patients were treated with chemotherapy alone.

### Definition of clinical end points

Achievement of CR was defined as a normocellular BM containing <5% leukemic blasts and showing normal maturation of all cell lineages. In addition, recovery of neutrophils 1500/μl and platelets >100 000/μl in peripheral blood was mandatory, as well as no evidence for circulating blasts and/or extramedullary leukemia. Relapse was defined as reoccurrence of >5% of leukemic blasts in BM, reappearance of circulating blasts or the development of extramedullary leukemia.^19^ Overall survival (OS) was defined as the time from diagnosis to death or date last known alive. Event-free survival (EFS) was defined as the time from diagnosis to relapse for patients who experienced a relapse, time to death for non-relapsed patients who did not survive or time to last follow-up for surviving patients who did not experience a relapse.

### Statistical analyses

Differences in clinical variables according to mutation status were investigated using Fisher's exact test for categorical variables and the Mann–Whitney *U*-test for continuous variables. OS and EFS probabilities were calculated using the Kaplan–Meier survival analysis and the differences in survival curves were compared using a two-sided log-rank test. Cox proportional hazard models were used to estimate hazard ratios for univariate and multivariate analyses for OS and EFS. To extract independent events, those with a *P*-value ⩽0.20 were analyzed using the forward stepwise model selection procedure. All statistical analyses were performed using SPSS 12.0.1 for Windows (SPSS Inc., Chicago, IL, USA). A two-sided *P*-value <0.05 was used to assess statistical significance.

## Results

### Detection of *FLT3*-ITD with fragment analysis

**Figure 1:**
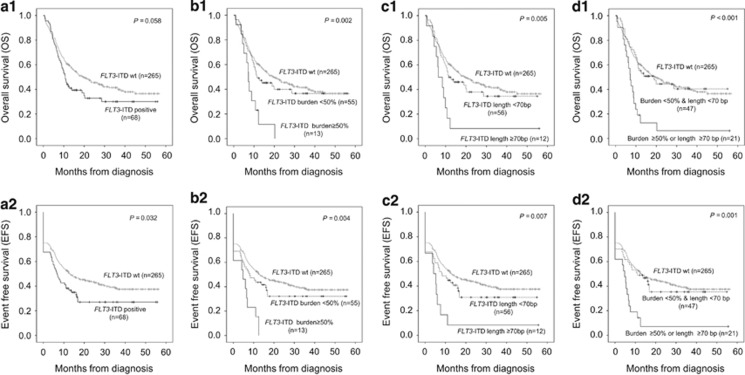
OS and EFS in the entire patients group according to the *FLT3*-ITD status. Presence or absence (**a**), mutant allele burden (**b**), mutant length (**c**) and both mutant allele burden and length (**d**).

The main characteristics of patients are summarized in Table 1. *FLT3-*ITD was identified in 73 of 363 adult AML patients (20.1%) and 71 patients (97.3%) had a single ITD and two patients had double ITDs at diagnosis. Presence of ITDs were associated with higher white blood cell count (median of 45.9 × 10^9^/l vs 5.3 × 10^9^/l; *P*<0.001) and higher peripheral blood blast percentage (median of 74 vs 24.5%, *P*<0.001), higher BM blast percentage (median of 86 vs 70% *P*<0.001). *FLT3*-ITD were more common in patients with normal karyotypes (frequency, 29.5 vs 10.6% *P*<0.001) and *NPM1* mutation (frequency 37.0 vs 2.4% *P*<0.001). CR rates were not significantly different between patients with or without *FLT3*-ITDs (*P*=0.214). OS was somewhat poorer for *FLT3*-ITD-positive patients (estimated median OS (95% CI) 10.7 months (8.7–12.7) vs 19.4 months (12.9–25.9)) (*P*=0.058) compared with *FLT3*-ITD-negative patients. EFS was poorer for *FLT3-*ITD-positive patients (estimated median EFS (95% CI) 6.6 months (3.8–9.4) vs 13.0 months (6.8–19.1)) (*P*=0.032; Figure 1a).

### Characteristics and clinical significance of *FLT3-*ITD mutant allele burden and length

#### *FLT3*-ITD mutant allele burden

The allelic burden of the ITD mutant in each patient ranged from 2.3 to 75.2% with a median of 32.9% (Table 2). According to the distribution of mutant allele burden and a previous study,^7^ we grouped patients with ⩾50% as high mutant allele burden. Thirteen patients (17.8%) were included in the high mutant allele burden group and 60 patients in the low mutant allele group (Supplementary Table S1). The mutant allele burden was associated with OS (estimated median OS of 7.2 months (5.7–8.6), 11.5 months (3.5–19.5) and 19.4 months (12.9–25.9); *P*<0.001) and EFS with estimated median EFS 4.8 months (0.0–11.8), 8.1 months (2.4–13.8) and 13.0 months (6.8–19.1); *P*=0.002) in *FLT3*-ITD high, low and wild type, respectively (Figure 1b).

#### *FLT3*-ITD mutant length

We next examined whether ITD length had an impact on clinical outcome. The length of the ITDs ranged from 16 to 150 base pairs (bp) with a median of 50 bp. The distribution of mutant length suggested two groups containing cases of less than 70 bp (*n*=57, 79.5%) and longer length with 70 bp or more (*n*=15). *FLT3*-ITD length was not significantly correlated with age, white blood cell count, peripheral blood blast percentage, BM blast percentage or ITD mutant allele burden. Treatment outcomes were related with ITD length. ITDs exceeding 70 bp in length were associated with decreasing OS, with an estimated median OS 6.4 months (2.7–10.1) compared with shorter ITDs (11.5 months (2.5–20.5)) and *FLT3* wild type (19.4 months (12.9–25.9; *P*=0.005)). Likewise, EFS tended to decrease in patients with long ITD with estimated median EFS as 4.2 months (1.5–6.9) compared with shorter ITDs and *FLT3* wild type, which had a median EFS of 8.1 months (2.7–13.5) and 13.0 months (6.8–19.1; *P*=0.007; Figure 1c).

#### Identification of *FLT3-*ITD poor prognostic group

As a high mutant allele burden (⩾50%) and long ITD length (⩾70 bp) were associated with worse clinical outcomes, we considered patients with either high mutant allele burden or long ITD length as one independent prognostic group. Therefore, we identified them as the *FLT3-*ITD poor prognostic group. Twenty four patients were included in the group because only four cases had both high allele burden and long ITD length. Notably, 22 of 24 patients died during follow-up (91.7%). The *FLT3-*ITD poor prognostic group demonstrated decreasing OS (7.2 months (5.4–9.0)) compared with the other *FLT3*-ITD-positive patients (low allele burden and short ITD length) (18.9 months (7.5–30.3)) and wild type (19.4 months (12.9–25.9); *P*<0.001) and EFS (4.8 months (2.4–7.2)) than the other *FLT3*-ITD-positive patients (11.0 months (7.4–14.6)) and wild type (13.0 months (6.8–19.1); *P*<0.001) (Figure 1d).

The *FLT3*-ITD high allele burden or long ITD length remained a significant adverse prognostic factor for both OS and EFS. Cox multivariate analysis, with the variables considered being age, sex, white blood cell count, National Comprehensive Cancer Network cytogenetic risk status and *NPM1* mutation (Table 3). The influence of *FLT3*-ITD high allele burden or long ITD length on clinical outcomes was remained even when the analysis was limited to patients receiving HSCT.

### Clinical significance of *FLT3-*ITD in cytogenetically normal AML

**Figure 2:**
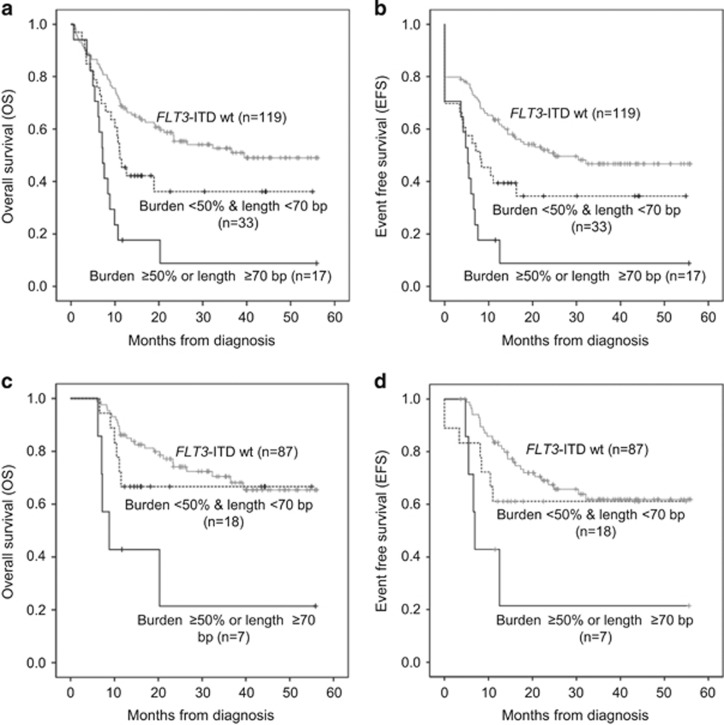
Comparison of OS and EFS according to *FLT3*-ITD status in CN-AML. Entire CN-AML (**a** and **b**) and patients treated with HSCT (**c** and **d**).

Next, the analysis was restricted to patients with cytogenetically normal (CN) AML (*n*=183) because the influence of *FLT3*-ITD is most strong in CN-AML, which is re-classified as poor-risk category when ITD is accompanied. In CN-AML, *FLT3-*ITD poor prognostic group (*n*=19) revealed inferior clinical outcome (median OS 7.2 months, *P*<0.001; median EFS 5.3 months, *P*<0.001) as like in entire patients. Interestingly, even patients with *FLT3*-ITD low allele burden and short length (*n*=35) showed poor OS (median 39.4 vs 11.1 months, *P*=0.037) and EFS (median 24.9 vs 8.1 months, *P*=0.044) when compared with *FLT3* wild-type patients. However, patients with *FLT3*-ITD low allele burden and short length did not show significantly different OS (*P*=0.329) and EFS (*P*=0.288) rates compared with *FLT3-*ITD wild-type CN-AML when the analysis was restricted in HSCT-performed CN-AML patients (*n*=112; Figure 2). These results indicated that the influence of the prognostic impact of *FLT3-*ITD was overcome when HSCT was performed except *FLT3-*ITD poor prognostic group.

### MRD monitoring using *FLT3*-ITD with fragment analysis

**Figure 3:**
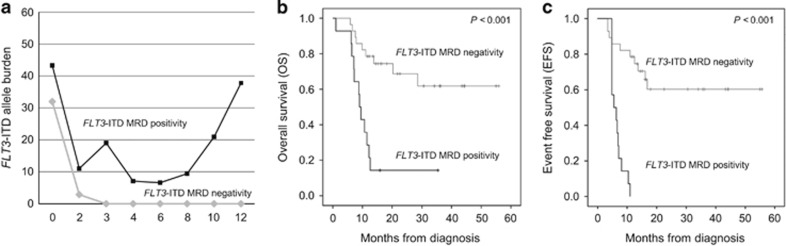
MRD group according to *FLT3*-ITD mutant allele burden after consolidation therapy. Serial follow-up *FLT3*-ITD mutant allele burden (**a**), OS (**b**) and EFS (**c**) according to MRD group.

Considerable effort has been directed at identifying molecular markers that can be used to detect residual disease or predict relapse at an earlier stage and lead to therapeutic intervention before overt hematological relapse occurs. Presently, the utility of *FLT3*-ITD mutant allele burden was evaluated by fragment analysis as a MRD marker. Forty two patients with *FLT3-*ITD were serially followed during treatment. *FLT3*-ITD negativity was demonstrated after consolidation therapy in 28 (66.7%) patients. Initial ITD allele burden (median 35.5%, 6.6–74.6% at diagnosis) was dramatically decreased after induction therapy (median 0%, 0–27.4%) and was not detectable after consolidation. Patients with detectable ITD by fragment analysis (MRD positivity, *n*=14) revealed transient decrement of ITD mutant allele level after induction (11.0±19.2%), which was increased after consolidation therapy (Figure 3). MRD negativity after consolidation therapy was also a valuable predictor of better OS (40.0 vs 12.4 months, *P*<0.001) and EFS (37.4 vs 4.8 months, *P*<0.001). Among patients who achieved hematological CR (*n*=36), nine had detectable ITD during follow-up. Of note, all nine were relapsed or died. The other 27 patients maintained *FLT3*-ITD negativity during the 12-month follow-up. Relapsed patients retained identical *FLT3*-ITD mutations that were harbored at diagnosis except for one patient who gained another new ITD at the time of relapse. In a patient with two ITD-positive mutants at initial diagnosis, a minor mutant with an allelic burden of 40.1% became the dominant mutant, with an allelic burden of 64.1% at the time of relapse.

## Discussion

*FLT3*-ITD heralds a poor prognosis in AML. However, the risk conferred by *FLT3-*ITD mutation remains to be determined whether specific characteristics, such as the allelic burden, length of the mutation or specific sequence as well as the presence of absence of ITD are related. It should be defined preferentially to draw up adequate therapeutic plan for each patient. The present study was performed with the two aims. The first was to determine the specific characteristics of *FLT3*-ITD associated with clinical outcomes. The second was to evaluate the fragment analysis, which is commonly used technique because of its convenience, is acceptable to test *FLT3-*ITD in routine laboratory.^20^

On the basis of the results from this study, the presence or absence of *FLT3*-ITD did not properly predict patient outcome. The mutant allele burden was more predictable for prognosis when the cutoff was determined as 50%. The 50% cutoff value is consistent with the observation that higher levels are indicative of the presence of biallelic disease, at least in some cells. The mutant allele burden in patients with leukemia is one of the most important features modulating the prognostic impact of the mutation.^7, 21, 22^ Prognostic relevance of *FLT3*-ITD allele burden might be based on biological grounds: highest *FLT3*-ITD corresponds to a homozygous state, usually because of a process of uniparental disomy, whereas a low ITD burden might have arisen in a minor subclone, perhaps occurring at a later stage of the leukemogenic process.^23^

This study also suggests that ITD length may be important in disease prognosis. We set the cutoff as 70 bp on the basis of the distribution of the length and the simulation of the effect on patients' outcome. These data suggest that the long ITDs are associated with a worse OS and EFS. Whether it is the length of the ITD or the involvement of specific residues remains to be clarified. It is possible that longer ITDs may involve multiple functional domains and more effectively disrupt the autoinhibitory regulation of the kinase activity.^11, 12^

Because the high mutant allele burden and long ITD length were proved to be poor prognostic factors, we considered patients with at least one of them as *FLT3*-ITD poor prognostic group. Only four cases overlapped, so that the number of cases in this group was nearly double compared with each group. Patients in the *FLT3*-ITD poor prognostic group had an inferior clinical outcome even though HSCT was performed, because relapse after HSCT occurs frequently.^24^ The prognostic factor might help guide early intervention with a strategy, such as FLT3 inhibition and/or alteration in graft versus host disease prophylaxis in patients at high risk of relapse.^25^

Little is known about the significance of a low allele burden and short length in *FLT3-*ITD mutation. Patients with low ITD allele burden and *NPM1* mutation were reported to show a similar prognostic impact to *FLT3* wild-type AML, whereas those with low ITD and wild-type *NPM1* had a higher risk of relapse and shortened OS than *FLT3* wild-type patients.^26^ To investigate the potential effect of low allele burden and short length of *FLT3*-ITD, we evaluated the prognostic impact of *FLT3-*ITD in restricted to CN-AML. Patients with *FLT3*-ITD low allele burden and short length had poorer OS and EFS compared with *FLT3* wild type. The prognostic impact of *FLT3*-ITD low allele burden and short length was overcome when HSCT was performed, whereas not in the *FLT3*-ITD poor prognostic group. These results support the view that comprehensive evaluation of *FLT3-*ITD mutation and cytogenetic risk group might help identify patients who benefit from early allogeneic HSCT.

CR after chemotherapy and post-transplant remission in AML patients has been assessed using BM morphology as well as hematologic recovery. Morphologic evaluations are insensitive and better methods are needed to assess MRD in AML patients. *FLT*3-ITDs may provide an adequate MRD marker for nearly 25% of AML patients especially in CN-AML.^27^ However, whether *FLT3* mutational status is an adequate marker for MRD has been questioned, given that *FLT3* status can change over time.^28, 29^ Although a relapsed patient with emerging new clone and a patient showing shift of dominant clone were identified in this study, such alterations are not common. When relapse occurs in patients harboring the *FLT3*-ITD mutation at diagnosis, the mutation is present in most cases. In addition, our results confirm that *FLT3* positivity after consolidation therapy predicts treatment failure or relapse. Therefore, molecular *FLT3-*ITD monitoring is useful to assess MRD in those patients. Nine patients showed detectable ITDs in hematologic CR stuatus, which was regarded as incomplete molecular CR status. In those patients, more intensive chemotherapy, including early HSCT can be a replaceable solution instead of routine consolidation protocol.

In the view of analytical technique, a standard PCR assay using primers that straddle the ITD mutation has been developed to detect length-altering mutations of ITD.^30^ Amplicons with a size greater than that of wild type and labeled with both fluorochromes are interpreted as positive for ITD mutation. The advantage of ITD assay using fragment analysis is that this method is able to detect the allelic burden of wild-type and mutant *FLT3*, multiple clonality and length of ITD simultaneously without extra labor. The detection sensitivity is about 5% of the mutant allele burden^30^ and we also observed a similar limit of detection (~3%). The limitation of this method for quantification is that the wild-type allele being amplified at a more rapid rate than the mutant allele during PCR. This PCR bias affects the accuracy of mutant allele burden, so that long ITD mutant is measured to lower level than expected. We tried to minimize this bias by the modification of PCR cycle and confirmed the accuracy of the method using standardized material resulted in excellent linearity and high concordance rate. Continuous *FLT3-*ITD monitoring in patients achieving molecular remission demonstrated that MRD-negative status retained during the 12 months follow-up. Therefore, fragment analysis is useful technique for MRD monitoring.

Finally, our findings indicate that *FLT3-*ITD poor prognostic group with high mutant allele burden or long ITD length is efficiently identified by quantitative fragment analysis. In addition, *FLT3*-ITD is quite useful for MRD monitoring to detect incomplete remission or early relapse in molecular level. This *FLT3*-ITD analysis is capable of guiding clinical decision making, potentially including the use of another treatment modality such as FLT3 inhibitors.

## Figures and Tables

**Table 1:** Baseline characteristics according to the *FLT3*-internal tandem duplication mutation

	FLT3 *wt*	FLT3*-ITD positive*	FLT3*-ITD burden <50% and length <70* *bp*	FLT3*-ITD burden ⩾50% or length ⩾70* *bp*	*Total*
Number	290	73	49	24	363
Age, years, median (range)	50 (15–85)	52 (18–79)	49 (22–75)	54.5 (18–79)	51 (15–85)
Gender, male (%)	155 (53.4)	36 (49.3)	27 (55.1)	9 (37.5)	191 (52.6)
Leukocyte	52.75 (3.1–449.0)	45.9 (1.4–317.6)	64.1 (1.4–317.6)	322.9 (3.1–302.0)	82.8 (3.1–449.0)
Hb	8.75 (3.5–14.4)	8.8 (4.3–16.1)	8.8 (4.3–16.1)	8.8 (5.1–15.7)	8.8 (3.5–16.1)
Platelet	57.0 (5.0–646.0)	56.0 (7.0–374.0)	55.0 (10.0–374.0)	64.5 (7.0–263.0)	57.0 (5.0–646.0)
PB blast (%)	24.5 (0–98)	74 (1–98)	68.0 (1–98)	76.5 (2–98)	31 (0–98)
BM blast (%)	70 (12–100)	86 (24–99)	90.0 (40–99)	83.0 (24–98)	75 (12–100)
					
*Karyotype (NCCN)*					
Intermediate risk	215 (74.1)	63 (86.3)	42 (85.7)	21 (87.5)	278 (76.6)
Normal karyotype	129 (44.5)	54 (74.0)	35 (71.4)	19 (79.2)	183 (50.4)
Adverse risk	74 (25.5)	9 (12.3)	6 (12.2)	3 (12.5)	83 (22.9)
*NPM1* mutation	7 (2.4)	27 (37.0)	16 (32.7)	11 (45.8)	34 (9.4)
					
*Treatment course*					
Untreated	25 (8.6)	5 (6.8)	2 (4.1)	3 (12.5)	30 (8.3)
Induction					
IDA/BHAC	123 (42.4)	29 (42.4)	17 (34.7)	12 (50.0)	152 (41.9)
IDA/ARA-C	104 (35.9)	33 (45.5)	24 (49.0)	9 (37.5)	137 (37.7)
LDARA/VP16	38 (13.1)	6 (8.2)	6 (12.2)	0 (0.0)	44 (12.1)
CR within two cycles of CTx	199 (68.6)	46 (63.0)	33 (67.3)	13 (54.2)	245 (67.5)
Relapse during CTx	33 (11.4)	13 (17.8)	8 (16.3)	5 (20.8)	46 (12.7)
					
*Post-remission therapy*					
Intensive chemotherapy	24 (8.3)	10 (13.7)	5 (10.2)	5 (20.8)	34 (9.4)
LDARA maintenance	17 (5.9)	4 (5.5)	4 (8.2)	0 (0.0)	21 (5.8)
Auto-HSCT	17 (5.9)	2 (2.7)	2 (4.1)	0 (0.0)	19 (5.2)
Allo-HSCT					
MSD	69 (23.8)	19 (26.0)	15 (30.6)	4 (16.7)	88 (24.2)
URD	49 (16.9)	10 (13.7)	8 (16.3)	2 (8.3)	59 (16.3)
FMT	30 (10.3)	6 (8.2)	4 (8.2)	2 (8.3)	36 (9.9)

Abbreviations: ARA-C, cytosine arabinoside; BHAC, *N*_4_-behenoyl-1-β-d-arabinofuranosyl cytosine; BM, bone marrow; bp, base pair; CR, complete remission; FMT, familial mismatched transplantation; Hb, hemoglobin; HSCT, hematopoietic stem cell transplantation; IDA, idarubicin; ITD, internal tandem duplication; LDARA, low-dose ARA-C; MSD, matched sibling donor; NCCN, National Comprehensive Cancer Network; PB, peripheral blood; URD, unrelated donor; VP16, etoposide; wt, wild type. The values represent median (range).

**Table 2:** *FLT3*-ITD mutant allele burden and length according to subgroup

FLT3*-ITD*	*Number*	*Mutant allele burden*	*Length*
Positive	73	32.9 (2.3–75.2)	49 (16–150)
			
*Allele burden*			
Low (<50%)	60	23.9 (2.3–47.6)	48 (16–150)
High (⩾50%)	13	53.2 (50.0–75.2)	50 (19–125)
			
*ITD length*			
Short (<70 bp)	58	34.7 (2.3–75.2)	40 (16–69)
Long (⩾70 bp)	15	23.7 (4.5–71.1)	97 (70–150)
			
*Allele burden/length*			
<50% and <70 bp	49	27.4 (2.3–47.6)	41 (16–69)
⩾50% or ⩾70 bp	24	49.3 (4.5–75.2)	78 (19–150)

Abbreviations: bp, base pair; ITD, internal tandem duplication.

**Table 3:** Multivariate analyses of OS and EFS in AML patients

	*OS*		*EFS*	
*Variable*	*HR (95% CI)*	P	*HR (95% CI)*	P
Age	1.032 (1.022–1.043)	<0.001	1.026 (1.016–1.037)	<0.001
*FLT3*-ITD poor prognostic group	2.947 (1.778–4.886)	<0.001	2.297 (1.398–3.773)	0.001
NCCN (karyotype)	1.708 (1.233–2.367)	0.001	1.596 (1.161–2.196)	0.004
Male	1.032 (1.020–1.042)	0.03	—	—

Abbreviations: AML, acute myeloid leukemia; OS, overall survival; EFS, event-free survival; HR, hazard ratio; *FLT3*-ITD poor prognostic group, *FLT3-*ITD allele burden ⩾50% or length ⩾70 bp; NCCN, National Comprehensive Cancer Network Guidelines Acute Myeloid Leukemia.
