# Supplementary material for: Quantitative fragment analysis of FLT3-ITD efficiently identifying poor prognostic group with high mutant allele burden or long ITD length
Source: Blood Cancer J. 2015 Aug 14;5(8):e336–. doi: 10.1038/bcj.2015.61 (PMC4558586; doi:10.1038/bcj.2015.61)
Supplement: Supplementary Table S1 [file bcj201561x1.doc]

**Supplementary Table S1.** Baseline characteristics according to the *FLT3*-internal tandem duplication mutation length and allele burden

|  | *FLT3* wt | *FLT3*-ITD burden Low (<50%) | *FLT3*-ITD burden High (≥50%) | *FLT3*-ITD length Short (<70 bp) | *FLT3*-ITD length  Long (≥70 bp) |
| --- | --- | --- | --- | --- | --- |
| Number | 290 | 60 | 13 | 58 | 15 |
| Age, yrs, median (range) | 50 (15-85) | 52.5 (22-79) | 52 (18-73) | 49.5 (18-75) | 55 (34-79) |
| Gender, male (%) | 155 (53.4) | 31 (51.7) | 5 (38.5) | 30 (51.7) | 28 (48.3) |
| Leukocyte | 52.75 (3.1-449.0) | 55.6 (1.4-317.6) | 43.9 (3.1-302.0) | 60.97 (1.4-317.6) | 30.28 (3.5-123.5) |
| Hb | 8.75 (3.5-14.4) | 8.9 (4.3-16.1) | 8.4 (5.1-11.8) | 8.7 (4.3-16.1) | 8.9 (6.9-15.7) |
| Platelet | 57.0 (5.0-646.0) | 53.5 (7.0-374.0) | 78.0 (22.0-263.0) | 56.5 (10.0-374.0) | 48.0 (7.0-149.0) |
| PB blast (%) | 24.5 (0-98) | 68.5 (1-98) | 81 (5-98) | 69 (1.0-98.0) | 76 (2.0-98.0) |
| BM blast (%) | 70 (12-100) | 86 (30-99) | 87 (24-98) | 90 (24-99) | 81 (30-98) |
| Karyotype (NCCN) |  |  |  |  |  |
| Intermediate-risk | 215 (74.1) | 52 (86.7) | 11 (84.6) | 49 (84.5) | 14 (93.3) |
| Normal karyotype | 129 (44.5) | 44 (73.3) | 10 (76.9) | 42 (72.4) | 12 (80.0) |
| Adverse-risk | 74 (25.5) | 7 (11.7) | 2 (15.4) | 8 (13.8) | 1 (6.7) |
| *NPM1* mutation | 7 (2.4) | 20 (33.3) | 7 (53.8) | 22 (37.9) | 5 (33.3) |
| Treatment course |  |  |  |  |  |
| Untreated | 25 (8.6) | 5 (8.3) | 0 (0.0) | 2 (3.4) | 3 (20.0) |
| Induction |  |  |  |  |  |
| IDA/BHAC | 123 (42.4) | 23 (38.3) | 6 (46.2) | 21 (36.2) | 8 (53.3) |
| IDA/ARA-C | 104 (35.9) | 26 (43.3) | 7 (53.8) | 29 (50.0) | 4 (26.7) |
| LDARA/VP16 | 38 (13.1) | 6 10.0) | 0 (0.0) | 6 (10.3) | 0 (0.0) |
| CR within 2 cycles of CTx | 199 (68.6) | 38 (63.3) | 8 (61.5) | 38 (65.5) | 8 (53.3) |
| Relapse during CTx | 33 (11.4) | 10 (16.7) | 3 (23.1%) | 10 (17.2) | 3 (20.0) |
| Post-remission therapy |  |  |  |  |  |
| Intensive chemotherapy | 24 (8.3) | 8 (13.3) | 2 (15.4) | 5 (8.6) | 5 (33.3) |
| LDARA maintenance | 17 (5.9) | 4 (6.7) | 0 (0.0) | 4 (6.9) | 0 (0.0) |
| Auto-HSCT | 17 (5.9) | 2 (3.3) | 0 (0.0) | 2 (3.4) | 0 (0.0) |
| Allo-HSCT |  |  |  |  |  |
| MSD | 69 (23.8) | 16 (26.7) | 3 (23.1) | 18 (31.0) | 1 (6.7) |
| URD | 49 (16.9) | 9 (15.0) | 1 (7.7) | 9 (15.5) | 1 (6.7) |
| FMT | 30 (10.3) | 4 (6.7) | 2 (15.4) | 5 (8.6) | 1 (6.7) |

Abbreviations: wt, wild type; ITD, internal tandem duplication; hemoglobin; PB, peripheral blood; BM bone marrow; IDA, idarubicin; BHAC, N_4_-behenoyl-1-β-D-arabinofuranosyl cytosine; ARA-C, cytosine arabinoside; LDARA, low dose ARA-C; VP16, etoposide; CR, complete remission; HSCT, hematopoietic stem cell transplantation; MSD, matched sibling donor; URD, unrelated donor; FMT, familial mismatched transplantation
